# Torsion of the epiploic appendix: An unusual cause of acute abdomen

**DOI:** 10.4103/0972-9941.33277

**Published:** 2007

**Authors:** Samik Kumar Bandyopadhyay, Mayank Jain, Shashi Khanna, Bimalendu Sen, Om Tantia

**Affiliations:** Department of Minimal Access Surgery, ILS Multipseciality Clinic,, Kolkata, India

**Keywords:** Acute abdomen, epiploic appendix

## Abstract

**Summary:**

Torsion of an epiploic appendix is a rare surgical entity. We present our experience in a thirty five year old female patient and a forty year old male patient.

**Materials and Methods:**

A 35 year old lady had presented with right iliac fossa pain of 2 days duration. Guarding and rebound tenderness was present over the area. Investigations showed mild leucocytosis and neutrophilia. Diagnostic laparoscopy revealed an inflamed epiploic appendix which was excised. Other intrabdominal organs were normal. A 40 year old male patient had presented with a history of recurrent, colicky, and paroxysmal right lower quadrant pain for 2 months. At laparoscopy, an inflamed torted epiploic appendix of the ascending colon was detected and excised. Other intrabdominal organs were normal.

**Results:**

Both the patients had an uneventful recovery and are asymptomatic at follow up of 10 and 7 months respectively. They have been followed up at 7 days, 4 wks and then 3 monthly.

**Discussion:**

The clinical presentation of an inflamed appendices epiploicae may be confusing. CT is helpful in disgnosis. Laparoscopy may be used to diagnose and treat the condition as well.

**Conclusion:**

Diagnostic laparoscopy is an useful tool for surgeons in assessing abdominal pain where the cause is elusive. It may be used to diagnose and treat torsion of an epiploic appendix effectively.

## INTRODUCTION

Torsion of an epiploic appendix is a rare surgical entity that presents with abdominal pain. In a series of 1320 cases of acute abdominal pain by Golash[1] only eight cases were due to acute epiploic appendagitis. The site of pain may vary according to the position of the inflamed appendage. Hence, it may mimic acute appendicitis, acute cholecystitis or acute diverticulitis.

We report two cases where the clinical presentations mimic acute appendicitis. It was only on diagnostic laparoscpy that gangrenous appendix epiploicae on the ascending colon was found in both the cases and proper management by excision of the gangrenous epiploicae undertaken.

So we suggest that though rare but epiploic appendigitis if present may closely mimic acute appendicitis and should be looked for if the appendix is found normal per-operatively.

This also highlights the importance of doing appendicectomy with concomitant diagnostic laparoscopy since many pathologies closely mimics appendicitis.

## CASE REPORTS

### Case 1

A 35-year-old female patient presented to the surgical outpatient department with a history of right lower quadrant pain for two days. She had a history of bouts of vomiting and associated low-grade fever for one day. There was no history of constipation, bleeding per anum or abdominal trauma. There was no major comorbidity. There was no history of dysuria or leucorrhoea or any menstrual disorder. On examination, there was guarding over the right iliac fossa and mild tenderness over the Mcburney's point. Rebound tenderness was elicited but Rovsing's test, Cope's Psoas Test and Cope's Obsturator tests were negative.

In view of the clinical presentation, she was provisionally diagnosed to be suffering from acute appendicitis. Laboratory and screening tests were asked for and parenteral antibiotics, analgesics etc were administered. She was allowed nothing per orally and intravenous fluids were administered.

Ultrasonogram was unremarkable but the laboratory results showed mild leucocytosis (11,500/cm) with neutrophilia (78%). Since the pain was not getting controlled, a decision to perform diagnostic laparoscopy under General Anesthesia and proceed further was taken.

During laparoscopy, the vermiform appendix was found to be normal. The ileocaecal junction was identified and distal ileum was examined for about 60 cm - the ileum was healthy, there was no mesenteric lymphadenopathy and Meckel's diverticulum was not detected.

**Figure 1:**
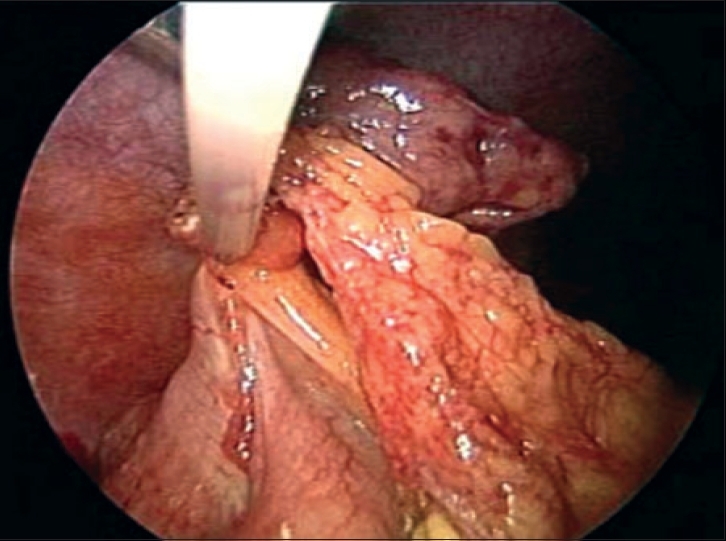
A gangrenous epiploic appendix

Examination of the ascending colon revealed a phlegmon of 4 cm size in the anterior surface. On gentle dissection, a swollen inflamed gangrenous epiploic appendix [Figure 1] with adjacent serosal inflammation was identified. The inflamed epiploic appendix had undergone gangrenous torsion. The epiploic appendix was excised with Ultrasonic Shears. Examination of the remaining intraabdominal organs was performed - no other disease process was identified. A local lavage was performed with warm normal saline, the lavage fluid was aspirated and the abdominal cavity was desufflated.

### Case 2

A 40-year-old male patient presented with a history of recurrent, paroxysmal, colicky right lower quadrant pain association with nausea and vomiting for two months. The last episode of pain was four days before the initial assessment; there was no history of trauma, constipation, diarrhea or bleeding per anum. On examination, there was marked tenderness over the right lower quadrant and Rovsing's test was positive. Provisionally, the patient was diagnosed as a case of recurrent appendicitis with an acute episode. Pelvic and lower abdominal Ultrasonogram was unremarkable and laboratory parameters were within normal limits.

A diagnostic laparoscopy was performed under general anesthesia. A normal-looking pelvic vermiform appendix was identified. The distal ileum and mesentry were also normal. Inflammation was identified in the ascending colon and adhesions to the greater omentum were present at the proximal ascending colon. On adhesiolysis with a Ultrasonic Shear an inflamed gangrenous, torsioned epiploic appendix was identified at the anteromedial part of proximal ascending colon. Excision of the appendage was performed and the abdominal cavity was desufflated after a local lavage with warm saline.

Both the patients had an uneventful postoperative period. The patients were discharged on the third and the fourth postoperative days respectively. Histopathological examination confirmed a gangrenous epiploic appendix in both patients.

## DISCUSSION

Appendices epiploicae are pedunculated structures lining the colonic extension. They are usually arranged in two axial rows from the caecum to the distal sigmoid colon. A normal adult human being usually has about 50-100 appendices epipoloicae. Primary epiploic appendagits is a rare condition,[1] in which torsion and inflammation of an epiploic appendix may cause localized abdominal pain. Epiploic appendagits usually occurs due to spontaneous venous thrombosis or torsion followed by hemorrhagic infarction, fatty necrosis, inflammatory reaction and subsequent peritoneal irritation. The disease commonly occurs in the adult population. The commonest site of epiploic appendagits is in the sigmoid colon followed by the caecum.

The site of pain may vary depending on the location of the appendage involved. Thus the disease may mimic acute appendicitis, acute cholecystitis or acute diverticulitis. However, in contrast to diverticulitis which shows mild smooth bowel thickening without lymphadenopathy, epiploic appendagits shows up as central areas of high attenuation and a hyper attenuated rim in proximity to the colon.[2]

Chronic torsion of an epiploic appendix has been reported to be associated with volvulus of a bowel segment with strangulated bowel obstruction.[3] Four deaths relating to the disease have also been reported in literature.[4] However torsion of an epiploic appendix is seldom diagnosed preoperatively. Interestingly, a significant number of patients with disease of epiploic appendix were found to have disorders of fat metabolism is a series from Russia.[5] Preoperative diagnosis of this condition is rarely made. At the present time, a laparoscopic exploration of peritoneal cavity will establish the correct diagnosis and the treatment can be provided during the same procedure.[6]

At operation, depending on the amount of torsion and / or inflammation several different findings may be possible - a phlegmon, a gaseous epiploic abscess, an infarcted epiploic appendix or a colonic mass.

Diagnostic laparoscopy is now considered a good diagnostic modality that offers an accurate assessment of this obscure pathology with the benefits of minimal risk and rapid recovery. In recent times, laparoscopic detection and treatment (by excision) has been reported from several centers. Though conservative treatment has been advocated by some centers,[7,8] a computerized tomographic scan (CT scan) was pivotal in formulating the principles of treatment.[9,10] In a developing country like ours where advanced scanning systems are not available everywhere, a diagnostic laparoscopy remains an accurate, safe and cost effective means of diagnosis which provides simultaneous therapeutic options at no increased expenditure.

Torsion of an epiploic appendix is a rare disease entity that may present with a confusing clinical spectrum and hence is difficult to diagnose. Diagnostic laparoscopy is a good treatment modality in such patients with abdominal pain but with an elusive diagnosis. Laparoscopy not only allows the surgeon to diagnose a torted epiploic appendix but allows the patient a benefit of simultaneous surgical treatment.
